# Mesenchymal Stem Cells Potentiate the Vasculogenic Capacity of Endothelial Colony-Forming Cells under Hyperglycemic Conditions

**DOI:** 10.3390/life12040469

**Published:** 2022-03-23

**Authors:** Hyunsook Lee, Yang-Hoon Huh, Kyu-Tae Kang

**Affiliations:** 1 College of Pharmacy, Duksung Women’s University, Seoul 01369, Korea; itzhyunsook@duksung.ac.kr; 2 Duksung Innovative Drug Center, Duksung Women’s University, Seoul 01369, Korea; 3 Electron Microscopy Research Center, Korea Basic Science Institute, Ochang 28119, Korea; hyh1127@kbsi.re.kr

**Keywords:** diabetes, hyperglycemia, vasculogenesis, endothelial colony-forming cells, mesenchymal stem cells

## Abstract

Many studies have demonstrated a reduced number and vasculogenic capacity of endothelial colony-forming cells (ECFCs) in diabetic patients. However, whether the vasculogenic capacity of ECFCs is recovered or not when combined with pericyte precursors, mesenchymal stem cells (MSCs), under hyperglycemic conditions has not been studied. Thus, we investigated the role of MSCs in ECFC-mediated vascular formation under high-glucose conditions. The ECFCs and MSCs were treated with normal glucose (5 mM; NG) or high glucose (30 mM; HG) for 7 days. The cell viability, proliferation, migration, and tube formation of ECFCs were reduced in HG compared to NG. Interestingly, the ECFC+MSC combination after HG treatment formed tubular structures similar to NG-treated ECFCs+MSCs. An in vivo study using a diabetic mouse model revealed that the number of perfused vessels formed by HG-treated ECFCs+MSCs in diabetic mice was comparable with that of NG-treated ECFCs+MSCs in normal mice. Electron microscopy revealed that the ECFCs+MSCs formed pericyte-covered perfused blood vessels, while the ECFCs alone did not form perfused vessels when injected into the mice. Taken together, MSCs potentiate the vasculogenic capacity of ECFCs under hyperglycemic conditions, suggesting that the combined delivery of ECFCs+MSCs can be a promising strategy to build a functional microvascular network to repair vascular defects in diabetic ischemic regions.

## 1. Introduction

Diabetic patients have a two- to four-fold increased risk of developing cardiovascular complications compared to non-diabetic people [1]. A prominent attribute of the cardiovascular complications in diabetic patients is endothelial injury and dysfunction, which can be caused by hyperglycemia, insulin resistance, oxidative stress, and inflammation [2,3]. Endothelial injury and dysfunction lead to the initiation and progression of atherosclerotic vascular diseases, which induce luminal narrowing, occlusion, and, finally, severe peripheral ischemia [4,5]. Peripheral ischemia results in end-organ damage, such as diabetic foot ulcers, retinopathy, neuropathy, and nephropathy. Thus, neovascularization to maintain blood perfusion can be a promising therapeutic strategy to prevent ischemic disability in diabetic patients.

Many different approaches have been tried to regenerate vascular networks in ischemic tissues. The delivery of the genes or proteins of angiogenic factors has been studied in depth to promote angiogenesis, but the results of clinical trials to date have not been fully successful. This may be due to overlooking the complex multi-step process of angiogenesis accomplished by many angiogenic factors. Over the last decade, building vascular networks using stem or progenitor cells has been a rapidly growing area of research aimed at reestablishing blood perfusion, thereby recovering the function of ischemic tissues. Clinically, autologous adult stem/progenitor cells, rather than embryonic stem cells, have emerged as a feasible strategy to achieve de novo vascular formation due to avoidance of the risk of teratoma formation [6] and host immune responses to allogeneic embryonic stem cells [7].

Endothelial colony-forming cells (ECFCs), also called late endothelial progenitor cells (late EPCs), are circulating endothelial precursors. These ECFCs are known to be recruited to the sites of injury/ischemia, where they promote vascular regeneration and repair [3]. Additionally, ECFCs can be isolated from human blood and bone marrow [8,9] and have robust proliferation capacity [10], which allow us to obtain a sufficient amount of cells for the purpose of cell therapy. Many research outcomes have strongly suggested that ECFCs are an autologous cell source for neovascularization to cure ischemic tissues. However, clinical studies have reported the reduced circulating number, as well as the impaired vasculogenic capacity, of ECFCs isolated from patients with type 1 and type 2 diabetes [3]. The deleterious effects of hyperglycemia on ECFCs has also been investigated in preclinical studies. For example, in vitro high-glucose culture conditions or a diabetic intrauterine environment diminish ECFC colony formation, self-renewal capacity, and tube formation [11,12]. These observations imply that the cardiovascular complications of diabetic patients are largely attributed to the defective vascular repair potency of ECFCs.

Blood vessels are composed of two cell types: endothelial and perivascular cells. ECFCs can differentiate to endothelial cells, thereby contributing to endothelial lumen formation [13]. Mesenchymal stem cells (MSCs) are pluripotent stem cells with the capacity to differentiate into perivascular cells when combined with endothelial cells, thereby contributing to vascularization [14,15]. We previously showed that the combined injection of human ECFCs and MSCs into the ischemic muscles of immunodeficient animals formed perfused human blood vessels more effectively compared to an injection of ECFCs alone [13,16]. Newly formed blood vessels by human ECFCs+MSCs maintained their structure for up to three months [17], and have an ability to reconnect with neighboring host vasculature within 3 days when transplanted in other sites [18].

Several approaches have been conducted to increase the circulating population and/or vasculogenic capacity of ECFCs under hyperglycemic conditions, but few studies have focused on the application of MSCs, pericyte precursors, to potentiate the vasculogenic capacity of ECFCs under hyperglycemia. Therefore, the purpose of our study was to determine the beneficial effects of MSCs on the vascularization of ECFCs under hyperglycemic conditions. We measured the cell viability, proliferation, migration, and tube formation of ECFCs after high-glucose (HG) or normal glucose (NG) treatment for 7 days. Although the cell viability, proliferation, migration, and tube formation of ECFCs alone were reduced in HG, the ECFC+MSC combination demonstrated a similar degree of tube formation between the HG and NG treatments. The in vivo vasculogenic capacity of HG-treated ECFCs+MSCs injected subcutaneously into diabetic immunodeficient mice was similar to that of NG-treated ECFCs+MSCs in normal mice. Our results demonstrate that MSCs can potentiate the vasculogenic capacity of ECFCs, which suggests that the combined delivery of ECFCs and MSCs could be a feasible therapeutic strategy to cure ischemic vascular diseases in diabetic patients.

## 2. Materials and Methods

### 2.1. Isolation and Culture of Human ECFCs and MSCs

The study protocol was approved by the institutional review board of Duksung Women’s University (IRB No. 2017-002-01). The endothelial colony-forming cells (ECFCs) were isolated from human peripheral blood provided from one normal adult male donor. Some CD31-coated magnetic beads (Invitrogen, Waltham, MA, USA) were used to isolate ECFCs from the adherent mononuclear cell fraction of blood, as described in a previous report [19]. The isolated ECFCs were expanded on 1% gelatin-coated plates (BD Biosciences, Franklin Lakes, NJ, USA) using endothelial cell growth medium (EGM-2; Lonza, Basel, Switzerland) without hydrocortisone, supplemented with 10% fetal bovine serum (FBS; Atlas Biologicals, Fort Collins, CO, USA) and 1% glutamine–penicillin–streptomycin (GPS; Gibco, Waltham, MA, USA). Mesenchymal stem cells (MSCs) isolated from one normal human adult bone marrow were purchased from Lonza and then cultured using MSC growth medium (Lonza) supplemented with 10% FBS and 1% GPS. The ECFCs and MSCs between passages 7 and 10 were used for all experiments.

### 2.2. High-Glucose Treatment

To mimic the hyperglycemic conditions of diabetic patients, the ECFCs and MSCs were cultured in each growth medium containing high glucose (30 mM; HG) for 7 days. The cells cultured in normal glucose-containing medium (5 mM; NG) were used as a normal glucose control.

### 2.3. Cell Viability Assay

The cell viability of the ECFCs and MSCs was determined by a 3-(4,5-dimethylthiazol-2-yl)-2,5-diphenyltetrazolium bromide (MTT) assay. The ECFCs or MSCs cultured in NG or HG for 7 days were harvested and added to a 96-well plate at 0.1 × 10^4^/100 μL/well, with each growth medium containing HG or NG. On days 1, 2, 3, 5, and 7, the cells were supplemented with MTT (0.5 mg/mL; Sigma-Aldrich, St. Louis, MO, USA) and incubated for 4 h. The formazan was dissolved with dimethyl sulfoxide (DMSO; Sigma-Aldrich), and the absorbance was measured at 650 nm. Quantitative graphs were made using the relative absorbance, which was calculated by dividing the absorbance of every other time point by the absorbance of NG on day 1.

### 2.4. Proliferation Assay

The proliferation ability of the ECFCs and MSCs were evaluated by counting 4′,6-diamidino-2-phenylindole (DAPI)-stained cells. The use of DAPI—a fluorescent stain that binds strongly to A-T-rich regions in DNA—is a traditional experimental protocol to count cells, even though many other alternatives to DAPI staining have been suggested recently. The ECFCs were seeded in a 24-well plate at a concentration of 0.4 × 10^4^/600 μL/well (the MSCs were seeded at a density of 0.2 × 10^4^/600 μL/well) and allowed to attach overnight at 37 °C. After 1, 3, 5, and 7 days of NG or HG treatment, the medium was removed and the cells were fixed with a 10% formalin solution (Sigma-Aldrich) for 30 min at room temperature. After fixation, the cells were washed one time with 1X phosphate-buffered saline (PBS; Corning, NY, USA), and then the mounting medium containing DAPI (Vectashield with DAPI; Vector Laboratories, Burlingame, CA, USA) was dropped into the middle of the well for nuclear staining. The DAPI-stained cells in images randomly taken per group from several independent experiments were counted using ImageJ software (NIH, Bethesda, MD, USA).

### 2.5. Migration Assay

The migratory function of the ECFCs and MSCs was evaluated by a scratch wound migration assay. The ECFCs or MSCs cultured in NG or HG for 7 days were harvested and added to a 24-well plate (ECFCs: 30 × 10^4^/1 mL/well; MSCs: 20 × 10^4^/1 mL/well) with each growth medium containing NG or HG. The cells were incubated at 37 °C for 16 h to allow the cells to attach to the bottom. After 16 h of incubation, the medium was changed to FBS-free medium containing NG or HG for serum starvation. After 16 h of incubation for serum starvation, the cell monolayer was scraped with a 200 uL pipette tip to generate scratch wound. The cells were rinsed with FBS-free medium to remove any floating debris. The scratch wound area was monitored at 0, 4, 12, and 24 h with an inverted microscope-connected camera (Koptic, Yongin, Gyeonggi-do, Korea). The magnitude of migration was evaluated by measuring the area of migrated cells (ImageJ software).

### 2.6. Tube Formation Assay

A tube formation assay was performed to determine the capability of the ECFCs to form a tubular structure in the absence or presence of MSCs. A 24-well plate was coated with ice-cold Matrigel solution (Phenol Red-Free; BD biosciences) and incubated at 37 °C for at least 30 min to allow the Matrigel to solidify. The ECFCs and MSCs cultured in each growth medium containing HG or NG for 7 days were harvested. ECFCs alone or ECFCs+MSCs were suspended in FBS-reduced medium (1%) containing NG, HG, or mannitol (30 mM; an osmatic control for HG), then added to the Matrigel-coated 24-well plate at a total of 15 × 10^4^/1 mL/well, which was incubated at 37 °C. The ratio of ECFCs and MSCs in the ECFC+MSC combination was 3:2 (9 × 10^4^:6 × 10^4^). The images of the tubular structures were taken by using an inverted microscope-connected camera (Koptic) at 6 h because the tube formation reached the maximum level at 6–8 h, and then the tubular structures were degraded at late time points [20]. The number and total length of tubes were analyzed with a total of nine randomly taken images of each group using ImageJ software.

### 2.7. Induction of Diabetes in Immunodeficient Mice

Animal experiments were conducted under a protocol approved by the Institutional Animal Care and Use Committee at Duksung Women’s University (approval no., 2014-016-002, 2016-003-007). Eight-week-old male immunodeficient mice (BALB/c-nude mice, Harlan Laboratories, Itingen, Switzerland) were fasted for 12 h, and then given streptozotocin (STZ) by intraperitoneal injection according to body weight (140 mg/kg) to induce diabetes. The mice were fasted for additional 3 h after STZ injection. Blood glucose was measured 3 days after STZ injection using a blood glucose meter (Accu-Check; Roche, Basel, Switzerland). Those mice with greater than 300 mg/dL of blood glucose were considered diabetic and were used for the experiments.

### 2.8. In Vivo Vasculogenesis Assay

To determine the in vivo vasculogenic capacity of human ECFCs+MSCs under diabetic conditions, we implemented a xenograft model with STZ-induced diabetic immunodeficient mice. The ECFCs and MSCs cultured in NG or HG for 7 days were harvested. The ECFCs and MSCs were mixed and suspended in ice-cold Matrigel (total 2.0 × 10^6^/150 μL). The ratio of ECFCs and MSCs in the combination was 3:2 (1.2 × 10^6^:0.8 × 10^6^). The ECFC+MSC-suspended Matrigel solution was injected subcutaneously into the dorsal area of a mouse using 25-gauge needle. The HG-treated ECFCs+MSCs were injected into the diabetic mice, and NG-treated ECFCs+MSCs were injected into normal mice. After 7 days, the perfused blood vessels formed by ECFCs+MSsC within the Matrigel implants were fluorescently labeled by the in vivo staining method, and then the Matrigel implants were harvested to analyze the perfused microvessel density.

### 2.9. In Vivo Staining by Tail Vein Injection of UEA-I and GS-IB_4_ Lectins

To identify the perfused functional blood vessels in Matrigel implants, we applied the in vivo staining method by tail vein injection of a mixture of rhodamine-conjugated *Ulex europaeus agglutinin I* (UEA-I; Vector Laboratoies), a lectin specific for human endothelium, and fluorescein isothiocyanate (FITC)-conjugated *Griffonia simplifolia* isolectin B_4_ (GS-IB_4_; Vector Laboratoies), a lectin specific for murine endothelium, as described previously [18]. Rhodamine-conjugated UEA-I and FITC-conjugated GS-IB_4_ were mixed into a 1 mM CaCl_2_ solution in saline. The lectin mixture (50 μg of each/100 μL/mouse) was injected intravenously 10 min before harvesting the Matrigel implants. The mice were euthanized and the implants were harvested, fixed in a 10% formalin solution overnight, incubated in 30% sucrose for another night, embedded in OCT, frozen, and cryosectioned.

### 2.10. Microvessel Density Analysis

Frozen sections of the impants of 12 μm in thickness were mounted using Vectashield with DAPI (Vector Laboratories). Perfused human or murine microvessels were identified as human- or murine-specific lectin-positive lumenal structures. Images for each sample were taken using a Zeiss LSM 700 Laser scanning microscope confocal equipped with a HBO 100 microscope illuminating system (four stable solid-state lasers: 405/444, 488, 555, and 639 nm) at room temperature. A 40×/1.25 or 20×/0.7 oil objective was used. The microvessel density (MVD) was analyzed by counting the vessels in each implant, reported as the vessel number per square millimeter.

### 2.11. Electron Microscopy

To observe the tubular structures formed by the ECFCs alone or ECFCs+MSCs in diabetic mice, the HG-treated ECFCs alone (2.0 × 10^6^/150 μL) or ECFCs+MSCs (1.2 × 10^6^ + 0.8 × 10^6^/150 μL) were suspended in Matrigel and injected subcutaneously into the diabetic immunodeficient mice. On day 7, the Matrigel implants were harvested and sequentially fixed with 2.5% glutaraldehyde and 1% osmium tetroxide on ice for 2 h, before being washed with PBS. The implants were then dehydrated in ethanol and propylene oxide series, embedded in an Epon 812 mixture, and polymerized in an oven at 70 °C for 24 h. The sections acquired from polymerized blocks were collected on grids, counterstained with uranyl acetate and lead citrate, and examined with the Bio-HVEM system (JEM-1400Plus at 120 kV and JEM-1000BEF at 1000 kV, JEOL, Tokyo, Japan).

### 2.12. Statistical Analysis

The values are expressed as the mean ± SEM. Since we had a small sample size, determining the distribution was important for choosing an appropriate statistical method. Thus, a Shapiro–Wilk test was performed, which did not show evidence of non-normality in any data set. Based on this outcome, the values were analyzed by ANOVA, followed by a Fisher’s least significant difference posthoc test for multiple comparisons, or were compared using Student’s *t*-test for paired comparisons (Origin software; OriginLab, Northampton, MA, USA). A value of *p* ≤ 0.050 was considered statistically significant.

## 3. Results

### 3.1. The Cell Viability and Proliferation Ability of ECFCs or MSCs in the HG Treatmennt

**Figure 1:**
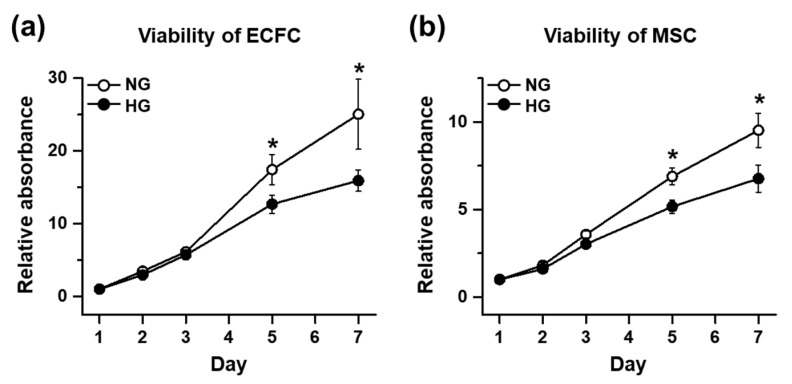
Cell viability of ECFCs or MSCs treated with NG or HG. Cells were cultured in growth medium containing normal glucose (5 mM; NG) or high glucose (30 mM; HG) for 7 days. The cell viability was determined by a 3-(4,5-dimethylthiazol-2-yl)-2,5-diphenyltetrazolium bromide (MTT) assay. (**a**) Quantitative graph of ECFC viability (*n* = 5; mean ± SEM). (**b**) Quantitative graph of MSC viability (*n* = 5; mean ± SEM). * Sigificant difference (*p* ≤ 0.05) between groups at the same time point.

**Figure 2:**
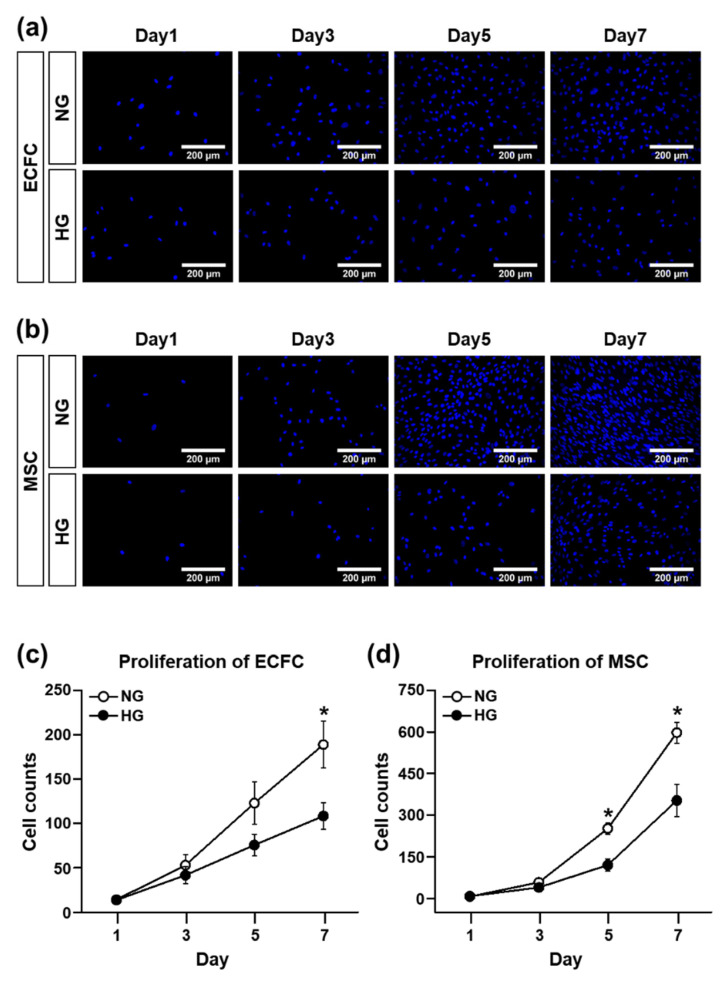
Proliferation of ECFCs or MSCs treated with NG or HG. Cells were cultured in growth medium containing normal glucose (5 mM; NG) or high glucose (30 mM; HG) for 7 days. The proliferation ability was determined by counting the DAPI-stained cells over time. (**a**) Representative images of DAPI-stained ECFCs at 1, 3, 5, and 7 days of NG or HG treatment (scale bar = 200 μm). (**b**) Representative images of DAPI-stained MSCs at 1, 3, 5, and 7 days of NG or HG treatment (scale bar = 200 μm). (**c**) Quantitative graph of ECFC proliferation (*n* = 4; mean ± SEM). (**d**) Quantitative graph of ECFC proliferation (*n* = 5; mean ± SEM). * Sigificant difference (*p* ≤ 0.05) between groups at the same time point.

The hyperglycemic condition of diabetic patients is known to reduce the circulating number, as well as impair the cellular functions, of endothelial colony-forming cells (ECFCs). The deleterious effect of hyperglycemia on ECFCs and mesenchymal stem cells (MSCs) was confirmed under our experimental conditions with normal-glucose (NG) or high-glucose (HG) treatment for 7 days. The results of the MTT assay demonstrated that the cell viability of the ECFCs was significantly reduced in HG compared with NG (Figure 1a). The HG-treated MSCs also showed reduced cell viability when compared to NG (Figure 1b). The proliferation ability of the cells was also evaluated by counting the DAPI-stained cells over time. The DAPI staining cannot differentiate live and dead cells; to solve this limitation, each well was washed once with PBS to remove the dead cells detached from the surface before staining with DAPI. Both ECFCs and MSCs showed reduced proliferation potential in HG compared to NG (Figure 2a–d).

### 3.2. Migration Ability of ECFCs or MSCs in the HG Treatmennt

**Figure 3:**
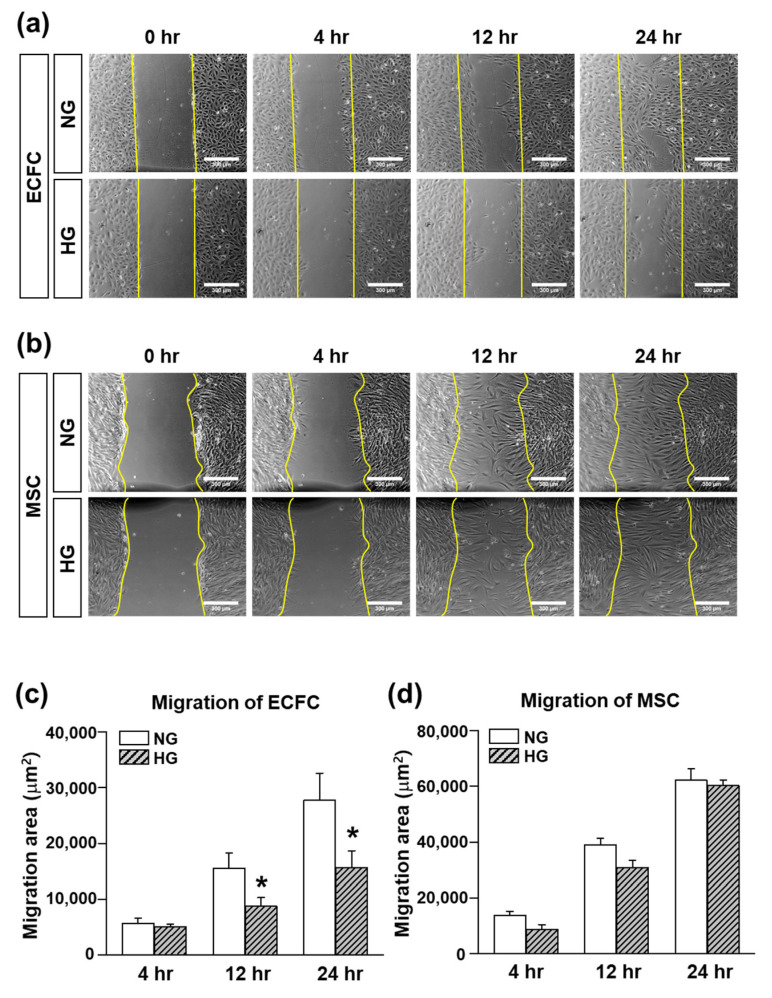
Migration of ECFCs or MSCs treated with NG or HG. Cells were cultured in growth medium containing normal glucose (5 mM; NG) or high glucose (30 mM; HG) for 7 days. The migration ability was evaluated by a scratch wound migration assay. (**a**) Representative images of migratory ECFCs at 0, 4, 12, and 24 h after scratching (scale bar = 300 μm). (**b**) Representative images of migratory MSCs at 0, 4, 12, and 24 h after scratching (scale bar = 300 μm). (**c**) Quantitative graph of the area covered by migrated ECFCs (*n* = 5; mean ± SEM). (**d**) Quantitative graph of the area covered by migrated MSCs (*n* = 3; mean ± SEM). * Sigificant difference (*p* ≤ 0.05) between groups at the same time point.

Cell migration is an essential process of vasculogenesis. The results of the scratch wound migration assay demonstrated that the migration ability of the ECFCs was significantly reduced in the HG compared to the NG treatment (Figure 3a,c). Interestingly, the migration ability of the MSCs was not different significantly between the NG and HG treatments (Figure 3b,d), suggesting less of an effect of the hyperglycemic conditions on MSC migration. Taken together, our data support previous clinical studies demonstrating reduced proliferation and migration abilities of ECFCs in diabetic patients, which may be related to the impaired repair process against diabetic vascular damage.

### 3.3. Tube Formation Capacity of ECFCs or ECFCs+MSCs in the HG Treatmennt

**Figure 4:**
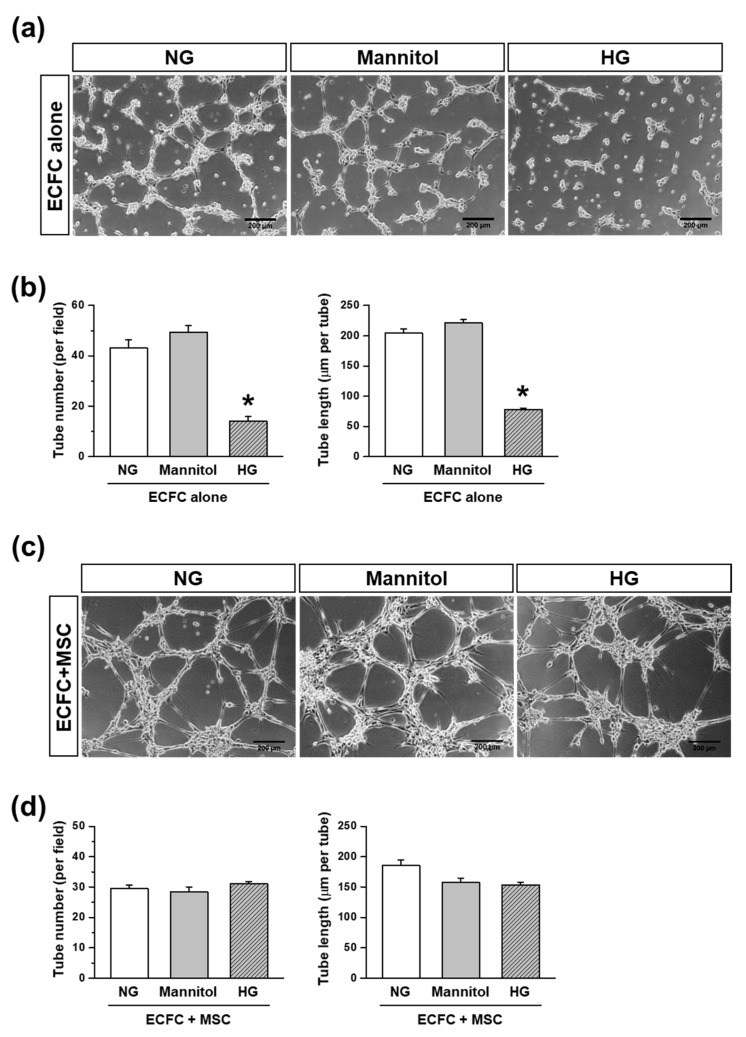
Tube formation of ECFCs or ECFCs+MSCs treated with NG or HG treatment. Cells were cultured in growth medium containing normal glucose (5 mM; NG), high glucose (30 mM; HG), or mannitol (30 mM; osmatic control for HG) for 7 days. Tube formation was analyzed at 6 h with 18 randomly taken images per group from several independent experiments (*n* = 3–5). (**a**) Representative images of the tube formation of ECFCs treated with NG, Mannitol, or HG (scale bar = 200 μm). (**b**) A quantitative graph of the tube number and total tube length formed by ECFCs treated by NG, Mannitol, or HG (*n* = 3–5; mean ± SEM). (**c**) Representative images of the tube formation of ECFCs+MSCs treated with NG, Mannitol, or HG (scale bar = 200 μm). (**d**) A quantitative graph of the tube number and total tube length formed by ECFCs+MSCs treated by NG, Mannitol, or HG (*n* = 3–5; mean ± SEM). * Sigificant difference (*p* ≤ 0.05) between groups.

To evaluate the effect of hyperglycemia on the vasculogenic capacity of ECFCs, the ECFCs were treated with NG or HG for 7 days. We conducted a parallel experiment with mannitol treatment (30 mM) to see whether the effect of HG was due to the hyperglycemic or hyper-osmotic conditions. The HG-treated ECFCs showed reduced tube numbers, as well as the total length of the tubes, compared to the NG-treated ECFCs (Figure 4a,b). Meanwhile, the mannitol-treated ECFCs showed a similar number of tubes and a similar total tube length to those treated with NG (Figure 4a,b), indicating that the high osmatic conditions did not have an effect on ECFC-mediate tube formation. These results suggest that the reduced vasculogenic capacity of ECFCs can be caused by the deleterious effect of hyperglycemia, which is the main characteristic of diabetes.

To determine the beneficial effect of combination with MSCs on vasculogenic capacity of ECFCs under hyperglycemic conditions, the ECFCs and MSCs were treated with NG or HG for 7 days. Then, the ECFCs and MSCs were harvested, mixed, and placed in Matrigel-coated wells. Excitingly, the tube formation by ECFCs+MSCs was comparable between HG and NG (Figure 4c). The quantified data also showed that there was no significant difference in tube number and total length of tubes between the groups (Figure 4d). Our results suggest that MSCs can potentiate the vasculogenic capacity of ECFCs under hyperglycemic conditions. Furthermore, the tubular structures formed by the ECFCs+MSCs seem to be thicker than those formed by the ECFCs alone (Figure 4a,c).

### 3.4. In Vivo Vasculogenic Capacity of HG-Treated ECFCs+MSCs in Diabetic Mice

**Figure 5:**
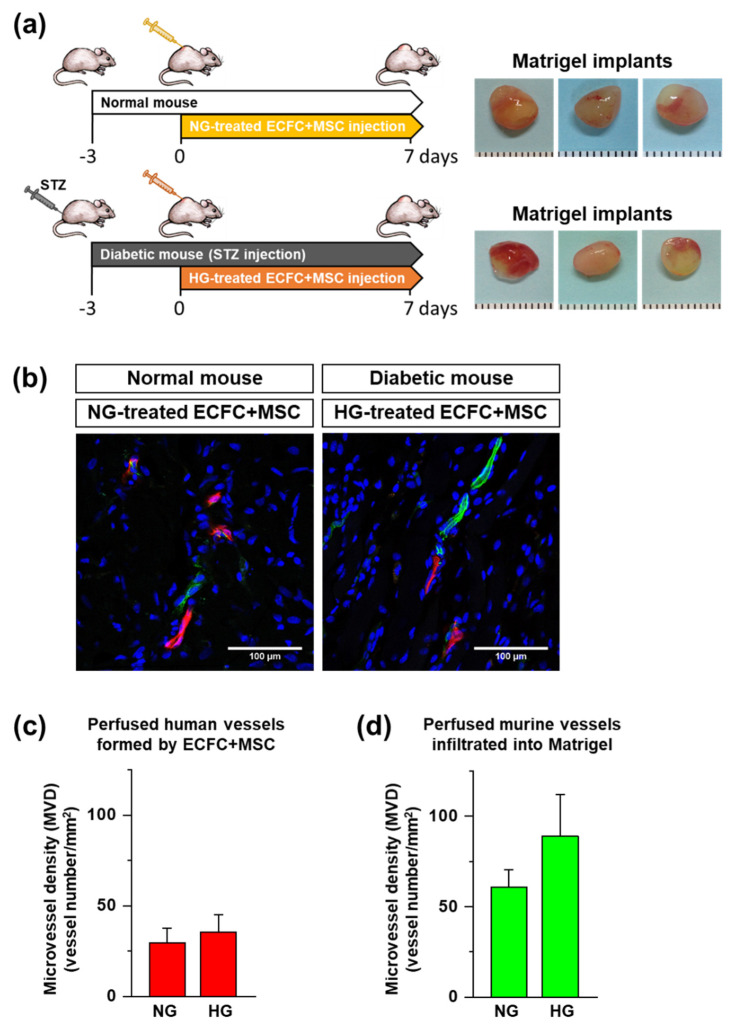
In vivo vasculogenic capability of ECFCs+MSCs under normoglycemic and hyperglycemic conditions. The HG-pretreated ECFCs+MSCs were suspended in Matrigel and injected subcutaneously into diabetic immunodeficient mice. To compare hyperglycemic and normoglycemic conditions, NG-pretreated ECFCs+MSCs were injected into normoglycemic healthy mice. Perfused human and murine vessels were identified by tail vein injection of a mixture of rhodamine (red)-conjugated UEA-I and FITC (green)-conjugated GS-IB_4_. (**a**) Experimental design and representative images of the harvested Matrigel implants. (**b**) Representative confocal images of lectin-labeled vessels within Matrigel implants on day 7 (scale bar = 100 μm). (**c**) A quantitative graph of the human microvessel density in the Matrigel implants (*n* = 7–12; mean ± SEM). (**d**) A quantitative graph of the murine microvessel density in the Matrigel implants (*n* = 7–12; mean ± SEM).

Previously, ECFCs isolated from diabetic patients have been shown to demonstrate an impaired response to chemotactic stimuli, reduced proliferative potential, and diminished ability to form vascular structures [3,21]. However, the in vivo vasculogenic capacity of HG-treated ECFCs in combination with MSCs has not been evaluated. The ECFCs and MSCs were treated with HG for 7 days, harvested, mixed together, suspended in Matrigel, and injected into streptozotocin (STZ)-induced diabetic immunodeficient mice. As the control group, NG-treated ECFCs+MSCs were injected into normoglycemic immunodeficient mice (see the experimental design in Figure 5a). The Matrigel implants were harvested on day 7 after cell injection. Perfused human and mouse vessels were visualized by tail vein injection of a mixture of human and mouse endothelium-specific lectins (rhodamine-conjugated UEA-I and FITC-conjugated GS-IB_4_) before harvesting the Matrigel implants. This in vivo staining method has merits in identifying perfused functional blood vessels in contrast to immature non-perfused vessels, and also in distinguishing human vessels (red) from murine vessels (green) (Figure 5b). As shown in Figure 5c, the perfused human vessels labeled by red fluorescence in the Matrigel implants were similar between diabetic and normal mice. Additionally, the perfused murine vessels labeled by green fluorescence were not different between groups (Figure 5d). This suggests that the reduced vasculogenic capacity of ECFCs in hyperglycemia can be enhanced by being combined with MSCs.

**Figure 6:**
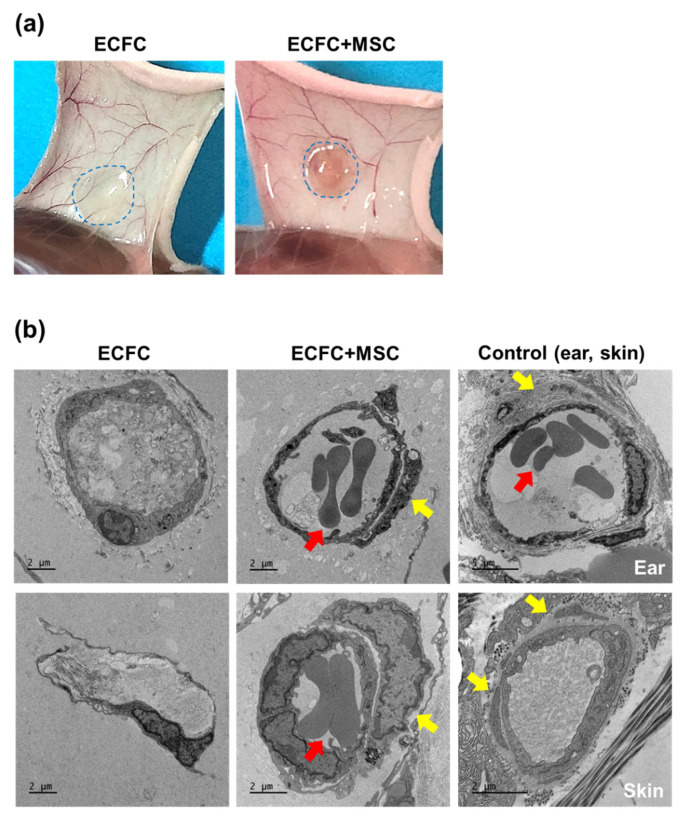
Electron microscopy images of ECFC- or ECFC+MSC-mediated blood vessels. HG-treated ECFCs alone (2.0 × 10^6^/150 μL) or ECFCs+MSCs (1.2 × 10^6^ + 0.8 × 10^6^/150 μL) were suspended in Matrigel and injected subcutaneously into diabetic immunodeficient mice. On day 7, the Matrigel implants were harvested and sectioned. The collected sections were examined with Bio-HVEM system (JEM-1400Plus at 120 kV and JEM-1000BEF at 1000 kV, JEOL, Tokyo, Japan). (**a**) Representative images of the Matrigel implants containing ECFCs alone or ECFCs+MSCs on day 7 after injection into diabetic mice. (**b**) Representative electron microscopy images of the tubular structures formed by ECFCs alone or ECFCs+MSCs (scale bar = 2 μm; *n* = 3). Red arrows indicate erythrocytes, and yellow arrows indicate pericytes. Ear and skin tissues were used to observe normal microvessel structures.

An injection of ECFCs into diabetic mice alone did not show red fluorescence-labeled perfused human blood vessels (data not shown). Thus, electron microscopy was applied to observe the tubular structures formed by ECFCs alone or ECFCs+MSCs within the Matrigel implants (Figure 6a). Ear and skin tissues were prepared to observe normal microvessel structures. Electron microscopy images revealed the obvious structural difference between ECFC- and ECFC+MSC-mediated blood vessels. As shown in Figure 6b, ECFCs+MSCs formed erythrocyte (red arrow)-filled blood vessels covered by perivascular cells (yellow arrows). Meanwhile, ECFCs alone formed only a prototype of vascular structures without coverage by perivascular cells (Figure 6b). Our results suggest that ECFCs+MSCs form perfused functional blood vessels, which may be due to the role of MSCs in stabilizing nascent vessel structures in a pericyte-like manner.

## 4. Discussion

The present study demonstrated a possible therapeutic approach to enhancing the vasculogenic potential of endothelial colony-forming cells (ECFCs) by combining them with mesenchymal stem cells (MSCs), perivascular cell precursors, for the vascular network re-establishment in diabetic ischemic tissues. The HG treatment reduced the cell viability, proliferation, migration, and tube formation of ECFCs. The HG also reduced the cell viability and proliferation of MSCs. Excitingly, HG-treated ECFCs+MSCs demonstrated a similar degree of tube formation ability to that of NG-treated ECFCs+MSCs. A diabetic mouse model was applied to determine the in vivo vasculogenic capacity of ECFCs+MSCs. The number of perfused human vessels formed by HG-treated ECFCs+MSCs implanted in diabetic mice was comparable to those formed by NG-treated ECFCs+MSCs implanted in normal mice. The electron microscopy images indicated that co-injected MSCs can enhance ECFC vascularization by stabilizing vascular structures in a pericyte-like manner. Our results suggest that MSCs potentiate ECFC-mediated vasculogenic capability even under hyperglycemic conditions; therefore, an injection of ECFCs+MPCs could be a feasible therapeutic strategy to build a functional microvascular network to repair diabetic ischemic tissues.

Diabetes is a complex metabolic disorder characterized by an impaired glucose metabolism with hyperglycemia. Chronic hyperglycemia causes endothelial dysfunction, which leads to diabetic vascular complications. The ECFCs, circulating endothelial progenitor cells, are known to be recruited to the injury/ischemic site, where they contribute to the endothelium repair and neo-vascular formation to maintain vascular homeostasis. Nonetheless, previous clinical and preclinical reports have demonstrated the decreased number and functional impairment of ECFCs isolated from diabetic patients. The reduced number and function of ECFCs are thought to be correlated with the severity of the vascular complications of diabetic patients [3], implying that ECFC dysfunction underlies the initiation and progression of vascular ischemic injury in diabetic patients.

The recovery of ECFC functions is considered one of the therapeutic options to repair endothelial defects in diabetic patients. Several approaches have been studied to enhance the ECFC functions in diabetic patients and animals. The administration of proangiogenic growth factors such as vascular endothelium growth factor (VEGF), basic fibroblast growth factor (bFGF), or a cocktail of them, has been shown to reverse ECFC dysfunction in diabetic patients [22]. Priming ECFCs with VEGF, bFGF, or platelet-derived growth factor (PDGF) also improves neovascularization in the wound-healing area of diabetic mice [23]. Evidence has been growing that the downregulation of microRNA, which can cause ECFC dysfunction, and the upregulation of microRNA 126 and 130α by encoding in ECFCs may be a new therapeutic approach [24,25]. The administration of SDF-1α and/or the expression of CXCR4 have also been investigated to improve the homing of ECFCs from circulation to injured tissues [26]. Although some promising results have been obtained from previous in vitro and in vivo studies, these may not fully recover the vasculogenic capacity of ECFCs. Vasculogenesis is a complex multi-step process involving not only several angiogenic growth factors, but also numerous cytokines, chemokines, proteinases, integrins, and cellular interactions. In particular, the cellular interaction between endothelial cells and pericytes are very important to form functional blood vessels. During the developmental stage, endothelial cell progenitors are responsible for the generation of new tubular structures, and pericyte precursors are responsible for stabilization of nascent blood vessels by wrapping tubular structures. This basic concept of vascular development inspired us to investigate the beneficial role of pericyte precursors, MSCs, in the vasculogenic capacity of ECFCs that is normally reduced under hyperglycemic conditions. In the present study, we first demonstrated that the combined treatment of ECFCs and MSCs, two vascular precursors, generated perfused microvessels more efficiently than ECFCs alone under hyperglycemic conditions. This effective method to regenerate a microvascular network to re-establish blood perfusion can be a promising therapeutic strategy to prevent ischemic disability of diabetic patients.

Previous studies have demonstrated the underlying mechanisms of vasculogenesis by ECFCs+MSCs. Fluorescent labeling of ECFCs and MSCs demonstrated that the ECFCs formed luminal structures, while the MSCs were located in the abluminal side surrounding the ECFC-lined vessels [13,27]. This integrated structure was clearly visualized by electron microscopy in the present study (Figure 6). Two vascular progenitor cells are known to interact with one another duing the vasculogenic process. The MSCs release angiogenic factors such as VEGF [13], which strongly activate ECFCs to generate new blood vessels. The MSCs also stimulate host myeloid cell recruitment [16,28], which potentiates blood flow recovery. The ECFCs serve not only as a building block of blood vessels, but also as paracrine mediators that regulate the regenerative potential of MSCs via PDGF-BB/PDGFR-β signaling [29]. Thus, the combined delivery of autologous ECFCs and MSCs can be a strong tool to regenerate the vascular network at the site where blood perfusion is needed. For clinical application, further studies are necessary to improve the isolation rate of ECFCs from patients, to improve the retention and survival rate of cells at the injection site, and to set up culture conditions without animal-derived ingredients of the culture medium such as fetal bovine serum.

In summary, hyperglycemic conditions negatively influence human ECFCs and MSCs. However, when applied as a combination of ECFCs+MSCs, MSCs enhance the vasculogenic capacity of ECFCs to form functional microvessels under in vitro and in vivo hyperglycemic conditions. Our findings indicate that a partnership between ECFCs and MSCs can be developed as a therapeutic strategy for vascular network re-establishment within the ischemic tissues of diabetic patients.

## Data Availability

The data supporting the conclusions of this article is included within the article.
